# Molecular signatures mostly associated with NK cells are predictive of relapse free survival in breast cancer patients

**DOI:** 10.1186/1479-5876-11-145

**Published:** 2013-06-12

**Authors:** Maria Libera Ascierto, Michael O Idowu, Yingdong Zhao, Hanif Khalak, Kyle K Payne, Xiang-Yang Wang, Catherine I Dumur, Davide Bedognetti, Sara Tomei, Paolo A Ascierto, Anil Shanker, Harry D Bear, Ena Wang, Francesco M Marincola, Andrea De Maria

**Affiliations:** 1 Infectious Disease and Immunogenetics Section (IDIS), Department of Transfusion Medicine, Clinical Center and FOCIS Center of Excellence, National Institutes of Health, Bethesda, MD, USA; 2 Department of Pathology, Virginia Commonwealth University, Massey Cancer Centre, Richmond, VA, USA; 3 Biometric research Branch, Division of Cancer Treatment and Diagnosis, National Cancer Institute, National Institutes of Health, Bethesda, MD, USA; 4 Weill Cornell Medical College (WCMC), Doha, Qatar; 5 Department of Microbiology & Immunology, Virginia Commonwealth University, Massey Cancer Centre, Richmond, VA, USA; 6 Department of Human Genetics, Virginia Commonwealth University, Massey Cancer Centre, Richmond, VA, USA; 7 Unit of Medical Oncology and Innovative Therapy, Istituto Nazionale Tumori Fondazione “G. Pascale”, Naples, Italy; 8 Department of Health Sciences and Centre of Excellence for Biomedical Research University of Genoa, Genoa, Italy; 9 Department of Surgery, Virginia Commonwealth University, Massey Cancer Centre, Richmond, VA, USA; 10 IRCCS Az.Osp.Univ., San Martino-IST Istituto Nazionale Ricerca sul Cancro, Genoa, Italy; 11 Laboratory of Lymphocyte Function, Department of Biochemistry & Cancer Biology, School of Medicine, Meharry Medical College, Vanderbilt-Ingram Cancer Centre, Vanderbilt University, Nashville, TN, USA; 12 Chief Research Officer Sidra Medical and Research Centre, Doha, Qatar; 13 Present Address: Infectious Disease and Immunogenetics Section (IDIS), Department of Transfusion Medicine, National Institutes of Health, Bldg 10, Room 1N224, 9000 Rockville Pike, Bethesda, MD 20892, USA

**Keywords:** Breast cancer prognosis, Molecular markers, Innate immunity, NK cells, Tumour relapse, Tumour microenvironment

## Abstract

**Background:**

Recent observations suggest that immune-mediated tissue destruction is dependent upon coordinate activation of immune genes expressed by cells of the innate and adaptive immune systems.

**Methods:**

Here, we performed a retrospective pilot study to investigate whether the coordinate expression of molecular signature mostly associated with NK cells could be used to segregate breast cancer patients into relapse and relapse-free outcomes.

**Results:**

By analyzing primary breast cancer specimens derived from patients who experienced either 58–116 months (~5-9 years) relapse-free survival or developed tumor relapse within 9–76 months (~1-6 years) we found that the expression of molecules involved in activating signaling of NK cells and in NK cells: target interaction is increased in patients with favorable prognosis.

**Conclusions:**

The parameters identified in this study, together with the prognostic signature previously reported by our group, highlight the cooperation between the innate and adaptive immune components within the tumor microenvironment.

## Background

Increasing evidence indicates that some patients with cancer can generate an adaptive immune response specifically directed against antigenic proteins expressed by tumors. In particular, an adaptive T cell response, which is composed of both cytotoxic CD8+ T cells (CTLs) and CD4+ T cells, can promote the secretion of cytokines such as interferon gamma (IFNγ) and tumor necrosis factor alpha (TNFα) generating an acute inflammation which results in expansion of cytotoxic CD8+ T cells, tissue destruction and control of cancer growth [1]. Accordingly, an “adaptive immune response” signature has been associated with improved outcomes in several tumour types [1-4]. Thus, in the context of tumor immune surveillance most emphasis has been placed on adaptive immune responses while the role of innate immune cells and their cross talk with adaptive immune cells as played a minor role. However, recent observations suggest that immune-mediated tissue destruction, which includes tumour regression, is dependent upon coordinate activation of immune effector genes expressed by cells of the innate and adaptive immune systems [1,5-8]. Recently, we have shown that the anti-tumour efficacy of Adoptive Cell Therapy (ACT) in the FVBN202 transgenic mouse model of breast carcinoma depended on the presence of tumour-specific T cells as well as activated Natural Killer (NK) cells and NKT cells [9]. Among cells of the innate immune system, the NK cells represent a subset of lymphocytes that can rapidly respond to the presence of tumour cells and participate in antitumor responses [10]. The effective function of NK cells is strictly dependent on the balance in the expression of activating and inhibitory receptors which are able to interact with ligands present on the target cells [11]. NK cells also express adhesion molecules able to interact with tumour cells or dendritic cells (DCs), resulting in NK cell cytotoxicity or enhanced DC activation, respectively. The anti-tumour effect of NK cells has been well documented especially in animal models showing that reduced NK cell activity leads to a high incidence of tumour occurrence and metastasis [12,13]. Tumour rejection in mice has been correlated with precursor frequency of both tumour-specific CD8+ T cells and NK effector cells that interact with tumour cells via activating receptor Natural Killer Group 2D (NKG2D)-mediated mechanisms [14]. In humans, while prognostic T cell signatures are widely established, the role of NK cells or other immune effector cells has been poorly appreciated. Only recently it has been observed that a decrease in the expression of the activating receptors NKG2D and DNAX accessory molecule 1(DNAM1) by NK cells is associated with tumour progression in breast cancer patients [15]. Similar observations were made in the tumour types such as chronic lymphocytic leukaemia (CLL) and gastrointestinal stromal tumours (GIST) [16,17], showing that an increased expression of Natural Cytotoxicity Receptors (NCRs) was associated with survival of patients. Recently, we reported that an increased expression of the immune function genes was associated with relapse-free survival in patients with breast cancer [18]. Using the same cohort of patients, we sought to determine prognostic value of the innate immune response characterized by a differential expression of molecular signatures mostly associated with activating signalling of NK cells and their crosstalk with target cells. Here, we showed that the expression of Natural Cytoxicity Receptor 1 (NCR1), considered the most accurate surface markers for human NK cell identification [19,20], together with molecules mostly associated with NK cells and involved in NK cells: target cells interactions, are associated with prolonged relapse-free survival in breast cancer patients. On the contrary, we found that differential expression of CD56 and CD16 in tumour specimens as well as the expression of Killer-cell Immunoglobulin-like Receptors (KIRs) transcripts failed to do so.

## Material and methods

### Clinical specimens

Patients experiencing either 58–116 months (~5-9 years) relapse-free survival or tumour relapse within 9–76 months (~1-6 years) following initial surgery were studied. Samples were collected from female breast cancer patients’ definitive surgery specimens. Specimens with unknown tumour stage, lost to follow-up or HER-2/neu borderline were not included in our analyses. Frozen tissues derived from 14 patients (7 who were relapse-free and 7 whose tumours relapsed) were used for microarray analysis. Among the samples screened by microarray, only 11 samples were also screened for RT-PCR because of lack of additional total RNA to analyse. Additionally 5 new samples were included in the RT-PCR analysis reaching a total group of 16 analyzed samples. Paraffin-embedded tissues derived from 9 patients including 4 relapse patients and 5 relapse-free patients were also used for immunohistochemistry (IHC). Patients used in each assay and detailed patients’ information are further described in Additional file 1: Table S1.

All the patients have signed an Informed Consent Form and approved the publication of this report and any accompanying images. The study was reviewed and approved by the Institutional Review Board (HM10920 and 2471-Tissue Acquisition System for Cancer Research) at Virginia Commonwealth University.

### RNA amplification, probe preparation, microarray hybridization

Gene profiling analysis was performed on a 36 k human oligo array as previous described [21].

Whole-genome human 36 K oligo arrays, representing 25100 unique genes of the Operon Human Genome Array–Ready OligoSet version 4.0 were printed in house using oligos purchased from Operon. The designed is based on Ensemble Huma Databasebuil NCBI-35c, with a full coverage on the NCBI human Refseq dataset (04/04/2005).

Reference for human arrays was obtained by pooling PBMCs from 6 normal donors.

Total RNA from tumour and reference samples were isolated and amplified into antisense RNA (aRNA) as previously described [22,23]. Both reference and test aRNA were directly labelled using ULS aRNA Fluorescent Labelling kit (Kreatech Diagnostics, Amsterdam, The Netherlands) with Hy3 and Hy5 respectively and co-hybridized to the slides. After 20 hours of incubation at 42°C, the arrays were washed, dried and scanned using the Agilent Microarray Scanner.

### Real-Time (RT)-PCR

Real Time-PCR was assayed by TaqMan^®^ Gene Expression Assays (Applied Biosystems, Foster City, CA) using an ABI PRISM 7900HT Sequence Detection System. Primers and probes were obtained from ABI (TaqMan Assays-on-Demand gene expression products, Applied Biosystems). To normalize the amount of source RNA, 18S transcript from the same sample was measured and used as internal reference. Each targeted transcript was validated using the comparative Ct method for relative quantification (ΔΔCt) reference to the amount of a common reference gene (18S) using PBMCs from healthy donors. The fold difference between relapse-free and progressing groups was calculated using the comparative 2–ΔΔCt [24].

### Statistical analysis

Microarray data were uploaded to the mAdb databank (http://nciarray.nci.nih.gov) and further analysed using Partek (St. Louis, Missouri, USA) and BRBArrayTools developed by the Biometric Research Branch, NCI (http://linus.nci.nih.gov/BRB-ArrayTools.html) [25]. The whole gene expression profile consisted of 25100 genes. Unsupervised analysis was performed for class confirmation using Stanford Cluster Program and TreeView software [26]. Gene ratios were average corrected across experimental samples and displayed according to uncentered algorithm. Functional gene network analysis was performed using the Ingenuity Pathway Analysis system (IPA).

RT-PCR data were analysed using MedCalc Software (Mariakerke, Belgium), StatGraphics Centurion (Warrenton,Virginia, USA) and BRBArrayTools; to determine the difference of mRNA expression among relapse and relapse-free groups Mann-Witney test using MedCalc Software and StatGraphics Centurion were considered. Survival analysis, survival risk prediction analysis and Support Vector Machine (SVM) were performed using BRBArrayTools [25]. Univariant Cox proportional hazards models were fit to test individual NK selected gene expression levels for association with survival (Wald test, two-sided). Kaplan-Meier survival curves were plotted for patients with expression levels above and below the median for each NK selected gene. Permutation p-values for significant genes were computed based on 10000 random permutations. Hazard ratio is considered as the ratio of hazards for a two-fold change in the gene expression level. It is equal to exp(b) where b is the Cox regression coefficient.

A survival risk prediction model was built using the supervised principal component method of Bair and Tibshirani [27]. A Cox proportional hazards model was fit to relate relapse time to the first two principal component linear combinations of expression levels from 8 significantly expressed genes screened by RT-PCR. In order to evaluate the predictive value of the method, leave-one-out cross-validation was used. A high relapse index value corresponded to a high predicted hazard of relapse with correspondingly poor predicted outcome. The cross validated Kaplan-Meier survival curves were plotted for cases predicted to have above average risk (relapse index above the median in the cross-validated model) and for cases predicted to have below average risk (relapse index below the median in the cross-validated model). A permutation analysis was performed in which the relapse data was randomly shuffled among cases 1000 times and the entire cross-validation process was repeated in order to assess whether the association of expression data to relapse data was statistically significant. The log-rank statistics was generated for those permutations.

Support vector machine (SVM) with linear kernel functions was used to develop the prediction model for distinguishing relapse-free patients and progressing patients.

In order to estimate how accurately the classes can be predicted by this multivariate class predictor, the leave-one-out cross-validated (LOOCV) misclassification rate as well as cross- validation ROC curve analysis were computed. Permutation p value for the cross-validated misclassification error rate was also calculated by randomly shuffling the class labels 1000 times and repeating the entire cross validation procedure.

Gene expression-based Outcome for Breast cancer Online (GOBO) tool (http://co.bmc.lu.se/gobo) [28], was used to perform *in silico* analyses and develop Kaplan-Meier analysis on for breast cancer patients (n = 115) derived from four publicly available breast cancer datasets of gene expression [29-32]. The results were stratified into the two quintiles based on gene expression level of NCR3(NKp30), NCR1(NKp46), CD96, CRTAM, DNAM1 and NKG2D.

### Immunohistochemistry (IHC)

Slides from paraffin-embedded tumour specimens were subjected to Dako automated immunostainer (Dako, Carpinteria, CA). Anti-human antibodies towards NCR1 (NKp46) (Abcam Ab14823, 1:100), LFA-1 (Abcam ab52895, 1:250) and CD1d (Abcam, Ab11076, 1:100) were used. The antigen retrieval was achieved using DAKO PT module at high PH. In order to circumvent the endogenous biotin activity, we used Dako Envision Dual Link System-HRP (Dako, Capinteria CA) in a two-step IHC technique, based on HRP labelled polymer which is conjugated with secondary antibodies. The labelled polymer does not contain avidin or biotin, thereby avoiding the nonspecific endogenous avidin-biotin activity in the sections.

## Results

### Differential expression of genes involved in NK signature in breast cancer patients with favourable prognosis

**Figure 1:**
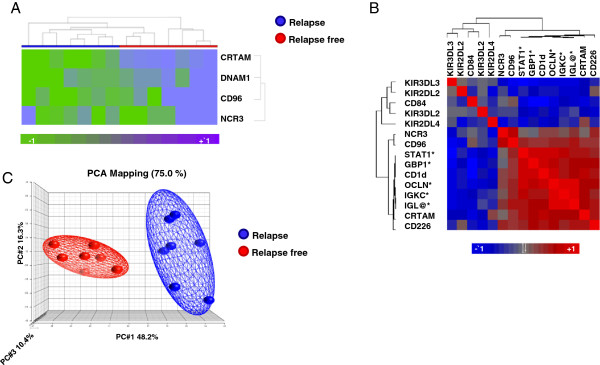
**Expression of genes involved in prognostic NK signature. A)** Unsupervised clustering based on genes involved in NK cell activation and interaction with tumour cells. **B)** Similarity matrix based on Pearson correlation assayed on a total of 15 including genes associated with NK, NKT and genes previously identified to predict breast cancer patients’ clinical outcome genes [6]. Genes previously identified are indicated with *. **C)** Principal Component Analysis based on 15 genes evaluated in 1B comparing relapse-free (red colour) and progressing (blue colour) patients.

Microarray analyses were performed on amplified RNA extracted from frozen tissues derived from 14 patients with either relapse free survival or tumour relapse (Additional file 1: Table S1). Unsupervised clustering performed on the filtered gene set (see material and methods) and based on DNAM1, NCR3(NKp30), CD96 and Class I–Restricted T-cell–Associated Molecule (CRTAM) expression signatures was able to spontaneously segregate the breast cancer patients in two distinct cohorts (Figure 1A). Unpaired Student’s t-test (p ≤ 0.001) was used to identify genes differentially expressed in relapse-free and relapsed groups. The cohort of the identified genes was further analyzed by IPA showing that genes involved in NK-DCs crosstalk (Additional file 2: Figure S1) and NK cells signalling (Additional file 3: Figure S2) were over-expressed in patients with favourable prognosis.

### Expression of genes involved in NK signature correlates with previously identified immune function gene signatures associated with favourable outcome

To test whether the expression of molecules mostly associated with NK cell signature correlated with the expression of 5 gene prognostic signature (STAT1, GBP1, OCLN, IGKC and IGL@) previously described by our group [6], a similarity matrix based on Pearson correlation was assembled considering 15 genes (KIR3DL3, KIR2DL3, KIR3DL2, KIR2DL4, CD84, NCR3(NKp30), CD96, CRTAM, DNAM1, CD1d, IGK@, GBP1, STAT1, IGLL5, and OCLN). We observed that the expression of genes involved in NK crosstalk with DCs and tumour cells (DNAM1, CRTAM, CD96), promoting NK cell activation (NCR3(NKp30)) or modulating NKT activity (CD1d) was strongly and positively correlated with that of the 5 previously identified markers of good prognosis. Conversely, genes known to encode for surface proteins that mediate MHC class-I mediated inhibition of NK cells, such as the KIRs receptors showed a negative correlation with the identified 5 immune gene signature (Figure 1B). Interestingly, there was an inverse correlation between the expression of the activating NK receptors, DNAM1 and NCR3, and CD84, a member of the signaling lymphocyte activation molecule (SLAM) immunoglobulin super family. Interaction of CD84 with SH2D1 provides activating stimuli for NK cells and T cells. On the other hand, the expression of CD84 has been reported to be critical for the survival of Chronic Lymphocytic Leukemia (CLL) [33] cells, suggesting an involvement in malignancy. Such controversial reports suggest that the biologic function of CD84 still remains to be determined.

Principal Component Analysis (PCA) based on the 15 genes set demonstrated that relapse-free patients (red colour) segregated apart from those with tumour relapse (Figure 1C), suggesting that the selected signature could be used to clearly differentiate the two cohort of patients. This confirmed previous observations suggesting that the balance between inhibitory and activating pathways in NK cells are finely regulated at the transcriptional level and activating pathways are important modulators of NK function in the tumour microenvironment.

### Increased expression of NK activating genes and NK adhesion molecules in patients without recurrence

**Figure 2:**
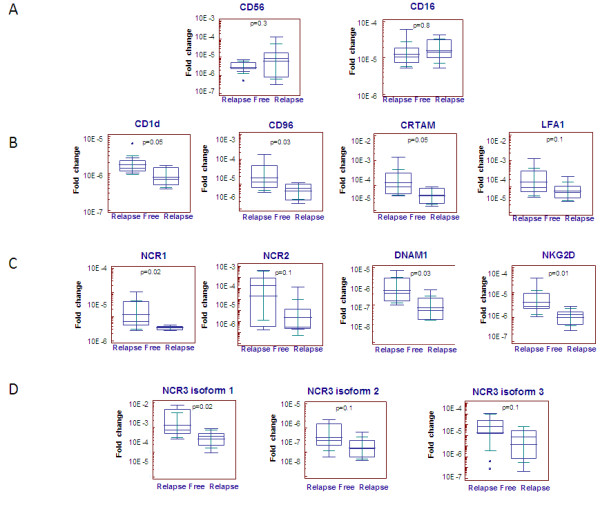
**RT-PCR analysis of relapse free and progressing patients. A)** Expression of NK identification markers ; **B)** Expression of genes encoding for molecules involved in NK crosstalk with target cells; **C)** Expression of genes involved in activation of NK functions; **D)** Expression of different isoforms of NCR3 (NKp30).

Results obtained from microarray analysis were further validated by conducting RT-PCR analysis. Because of the lack of additional total RNA, only 11 out of 14 samples previously screened by microarray analysis, were used for the RT-PCR screening. In addition to 11 samples, 5 independent samples, not previously screened by microarray analysis, were included in the analysis. Total RNA derived from the specimens of the16 patients were evaluated for the expression of 4 adhesion molecules which although not only expressed by NK cells are mostly involved in NK-target cells interaction (CRTAM, CD96, CD1d and Leukocyte Function-associated Antigen 1-LFA-1). Also genes involved in NK cell identification and activation were investigated (CD56, CD16, NKG2D, DNAM1). As already mentioned the above molecules are mostly associated with NK cells identification, activation and function, but they can be also shared with a fraction of other immune cells (e.g. T cells, NKT). Therefore 3 additional genes (NCR1(NKp46), NCR2(NKp44), NCR3(NKp30)) mostly restricted to NK cells [19] were screened. Intrigued by the fact that the expression of the 3 different isoforms of NCR3(NKp30) was differently associated with prolonged survival of patients with gastric GISTs [16], we performed quantitative RT-PCR using NCR3(NKp30) primers specific for the three isoforms, in order to investigate whether the expression of different isoforms of NCR3(NKp30) was differentially associated with the clinical outcomes also of individuals with breast cancer. Furthermore, we studied the expression of Forkhead box P3 (FOXP3), usually associated with the presence of T regulatory cells (Tregs) known to inhibit the activity of NK cells and to promote immune suppression function [34]. Based on differential expression of CD56 and CD16, NK cells did not vary between relapse free and progressing groups (p value = 0.3 and 0.8, respectively) (Figure 2A). However, tumours from patients with no recurrence were characterized by increased expression of genes involved in the interaction between NK cells and dendritic cells or tumour cells such as CD1d, CRTAM and CD96 (Figure 2B) and genes involved in activating signalling of NK cells such as NCR1(NKp46), DNAM1 and NKG2D (Figure 2C). Although all the three isoforms of the activating receptor NCR3(NKp30) are showed to be over expressed in the relapse-free group, only isoform1 was found to be significantly different (Figure 2D). Although not statistically significant, the activating receptor NCR2(NKp44) and the adhesive molecule LFA-1 were increased in the relapse free group (Figure 2B-2C).

Interestingly, RT-PCR results also highlighted a positive correlation between the expression of FOXP3 and favourable prognostic NK cells signature (Additional file 4: Figure S3A). One of the limitations of the current analysis is that the RT-PCR screening was mostly conducted on specimens already screened by microarray analysis and only 5 independent specimens were used. Thus, we cannot conclude that the differences observed are not biased by the individual genetic characteristics of the tumours; in order to widely validate the obtained results additional breast specimen’s collection is currently ongoing.

### Lack of prediction of breast cancer patients based on KIRs expression

Together with the 12 genes mentioned above, all 16 patients were also screened by RT-PCR for the expression of KIR2DL3, KIR3DL3 and KIR2DL2. The obtained data showed that expression of KIRs was not significantly different in groups with differential survival outcome (Additional file 4: Figure S3B) suggesting that genes involved in activating rather than inhibitory function of NK cells should be considered a potential predictive signature for better survival in breast cancer patients.

### Increased percentage of CD1d + and LFA + tumour cells in patients without recurrence

Protein validation analysis was performed by IHC using a set of 9 validation samples (Additional file 1: Table S1). Low levels of protein expression didn’t allow a clear detection of the NK specific marker NCR1 (NKp46) in both relapse-free and progressing patients (data not shown). Instead, tumour specimens of patients with relapse-free survival showed a higher percentage of CD1d + cells. Moreover a higher percentage of LFA-1+ cells was observed in patients with better outcome (Additional file 4: Figure S3C). The presence of CD1d and LFA on NKT cells and a small population of CD8+ cells, together with the lack of detection of Nkp46 signalling, didn't highlight a specific involvement of NK cells in favourable prognosis, at protein level. Although we consider this as a limit of our study, since we are looking for molecular signatures that may be associated with the potential triggering rather than with identification of individual cells involved with favourable outcome, the absence of additional IHC information can be considered a minor obstacle. In addition, if we consider that the screening of NK cells by IHC has always been centre of debate for lack of specificity and/or lack of protein detection, a molecular screening might seem more appropriate.

### NK cell signature predict patients outcome based on support vector machine prediction model

To test whether the genes screened by RT-PCR could be used to predict breast cancer patients’ outcome, SVM was used to develop the prediction model for distinguishing relapse-free patients and progressing patients. Among all the genes screened by RT-PCR, only 8 genes (DNAM1, CD96, CD1d, NKG2D, FOXP3 and NCR1(NKP46), NCR3(NKP30) isoform1 and CRTAM) with significantly difference p ≤ 0.05 between the two cohorts of patients were used in the following analysis.

**Figure 3:**
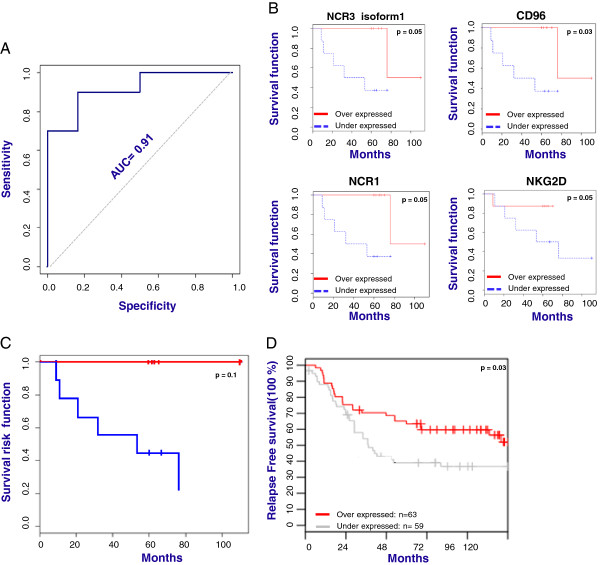
**Prediction models associated with survival and clinical outcome of breast cancer patients. A)** Cross-validation ROC curve analysis based on 8 genes (NCR1(NKP46), NCR3(NKp30) isoform1, NKG2D, CRTAM, DNAM1, CD96, CD1d) significantly and differentially expressed in relapse free and progressing patients based on RT-PCR analysis; **B)** Survival analysis based on the same group of genes. In the figure are reported only Kaplan Mayer curves of 4 genes with significant p value; **C)** Survival risk prediction analysis based on the same group of genes. **D)** Kaplan-Meier analysis, using RFS as endpoint, for breast tumors (n = 115) stratified into the two quintiles based on gene expression level of NCR1(NKP46), NCR3(NKp30), NKG2D, DNAM1 and CD96.

LOOCV analysis showed that the combination of the selected genes was able to predict with an accuracy of 81% patients’ outcome with 90% sensitivity and 66% specificity (Table 1). Based on 1000 permutations, the support vector machines classifier had p-value of 0.004 confirming that among the screened markers, the genes involved in NK cells function are significant predictors of breast cancer outcome. The same results were confirmed by Cross- Validation ROC curve analysis resulting in Area Under the Curve (AUC) equal to 0.91 (Figure 3A).

**Table 1:** Performance of the support vector machine classifier

**Class**	**Sensitivity**	**Specificity**	**PPV**	**NPV**
0	0.9	0.667	0.818	0.8
1	0.667	0.9	0.8	0.818

Support vector machine predictor was built as a linear function of the log-ratios of the expressions of 8 genes screened by RT-PCR. According to our LOOCV results, the 8 selected genes were able to predict with an accuracy of 81% patients’ outcome with 90% sensitivity and 66% specificity.

### Survival analysis and survival risk prediction model based on NK cell signature

In order to evaluate whether the molecular expression of genes differentially expressed among progressing vs. relapse-free groups could predict the survival of breast cancer patients, we performed by BRB array tools a survival analysis based on Cox proportional hazard models. Among the 12 genes screened by RT-PCR, only the 8 genes with significant differences among the two cohorts of patients were used in the following analysis.

Survival was related to each gene testing the hypothesis that survival time is independent of the expression level for that gene. A gene list was created based on the resulting p values which were challenged by a multivariate permutation test to control for the number or proportion of false discoveries. The results showed that the expression of CD96, NKG2D, CD96 and NCR1(NKp46) were significantly associated with survival of breast cancer patients (Figure 3B-Additional file 5: Table S2). Furthermore, survival risk prediction analysis demonstrated that the combined expressions of the selected genes was associated with decreased risk of disease progression, although the limited number of patients used in the analysis didn’t allow the permutation p-value of the log-rank test statistics between risk groups to be significant (p = 0.1, Figure 3C). In order to test whether the described result was associated with the age of patients or with their status of Estrogen Receptor (ER), Progesterone Receptor (PR), and Human Epidermal Growth Factor Receptor 2 (HER2) status rather than being related to the expression of NK cell signature described above, we performed a survival risk analysis using age, ER, PR and HER2 status as covariants. The results didn’t show an association between decreased risk of disease progression linked with the ER, PR and HER2 expression nor with age (Additional file 4: Figure S3D). In order to determine whether small number of samples may affect our results, by using GOBO tool [28] we conducted survival analysis on a larger cohort of breast cancer patients (n = 115) derived from four publicly available breast cancer datasets of gene expression [29-32]. Kaplan Meyer curves showed a significantly (p < 0.03) better survival for patients that showed higher expression of the NCR3 (NKp30), NCR1(NKp46), CD96, CRTAM, DNAM1 and NKG2D (Figure 3D).

## Discussion

The relevance of anti-tumour immune responses as modulators of cancer growth is increasingly recognized and most emphasis has been placed on adaptive immune responses. Yet, cross talk between the innate and adaptive immune systems may be a prerequisite for tumour rejection as recently described [1,8,35-37]. NK cells are proposed to be one of the major cells of the innate immune system involved in anti-tumour protection [37]. In human, it has been shown that the presence of intratumoral NK cells is positively correlated with favorable prognosis in colorectal, renal, lung cancer and hepatocellular carcinoma [38-41]suggesting that NK cells may represent a potential prognostic marker [42]. However, in the majority of these studies NK cells have been detected according to the expression of CD57 or CD56 markers which inaccurately identify NK cells since they are also expressed by other tumor-infiltrating lymphoid cells. Thus the involvement of NK cells in tumor immune surveillance has always been in the centre of debate.

Recently the advent of high throughput technology, mostly based on gene expression profiling has shown the relevance of the prognostic infiltration of NK cell markers at the tumor site.

A recent meta analysis conducted on a large publicly available set of microarray data from primary breast tumours suggested that breast cancer displayed variable expression of ligands for NK cell receptors. In particular, NKG2D-ligands and DNAM1-ligands, known for their NK cell activating function, were found to be widely expressed across all breast cancer subtypes [43]. The same group reported that expression of activating NK cell receptors decreased while expression of NK cell inhibitory receptors increased during breast cancer progression [15]. However, a comparative analysis of the expression of these markers in primary tumours directly linked to relapse-free survival has not been performed.

In the present study, we used gene expression profiling, RT-PCR and IHC analysis to detect the expression of activating and adhesive NK cell receptors in primary breast cancer specimens derived from patients who experienced either 58–116 months (~5-9 years) relapse-free survival or developed tumour relapse within 9–76 months (~1-6 years) following surgical resection.

Our results showed that the expression of the genes CD96, CD1d, CRTAM, LFA-1 and DNAM1, involved in the interaction of NK cells with target cells such as tumour cells or DCs, increased in patients with favourable prognosis. NK cells and DCs can exchange bidirectional activating signals in a positive feedback loop. DCs can support innate immunity by promoting the production of cytokines and cytotoxicity of NK cells against cancer. Reciprocally, NK cells have been shown to play immunoregulatory ‘helper’ functions, being able to activate DCs and to enhance their ability to produce pro-inflammatory cytokines during T cell responses [44]. The receptors CD96, CRTAM and DNAM1 promote adhesion of NK cells to target cells expressing the Poliovirus Receptor (PVR or CD155) [45,46]. This will in turn stimulate cytotoxic function of the NK cells and CD8+ T-cell secretion of IFN-γ in vitro as well as NK cell–mediated rejection of tumours in vivo. Also LFA-1 is critical for NK cell cytotoxicity as it mediates NK-cell binding to intercellular adhesion molecule1 (ICAM1) on target cells, generating activating signals that lead to polarization of the actin cytoskeleton and cytotoxic granules followed by perforin and granzymes exocytosis [47]. Besides its role in the crosstalk with target cells, DNAM1 together with the NKG2D, is considered one of the most important activating receptors expressed on NK cells. However, the molecules CD96, CRTAM and LFA1 may be expressed by activated CD8+ and CD4+ T cells as well as monocytes [19,48]. DNAM1 or NKG2D may also be expressed by T cells. However, it should be noticed the expression of these molecules was positively and significantly associated (R value > 0.86) with the transcription of activating receptors NCRs, considered mostly restricted to NK cells [19,49,50] . In addition, the expression of NCR1 (NKp46), considered the most accurate marker used for NK cell identification, was observed to be significantly increased in patients with favorable outcome ( p value 0.02) and was significantly associated with better survival of the patients (p value 0.03).

Although the number of patients screened for the breast cancer transcriptomic study was small, survival analyses performed on a larger number of breast cancer patients (n = 115) derived from four publicly available breast cancer datasets of gene expression [29-32] revealed a significantly (p < 0.03) better survival for patients that showed higher expression of the NCR3 (NKp30), NCR1 (NKp46), CD96, CRTAM, DNAM1 and NKG2D. This suggests that, together with cells of adaptive immune system, NK cells might play an important role in the context of tumor immune surveillance. The absence of detection of NCR1 (NKp46) by IHC didn’t allow us to validate at protein level a differential expression of NKp46 in patients with widely diverging outcomes. Although we consider this as a limit of the present study, it is important to noticed that the evaluation of NCR1 (NKp46) expression by IHC, as well as the evaluation of the NCR2 (Nkp44) and NCR3 (NKp30), has always been centre of debate for lack of an appropriate detection.

Thus a molecular screening is preferred and might be more accurate and useful in clinical practice.

Our results also showed that the expression of KIRs transcripts including KIR2DL3, KIR3DL3 and KIR2DL2 was negatively and significantly correlated with NCRs expression (R value < -0.7) and was not significantly different in patients with widely diverging outcomes. This evidence suggests that the balance between inhibitory and activating pathways in NK cells is finely regulated at the transcriptional level. Thus, molecules involved in activating signaling of NK cells, rather than in inhibitory one, might be considered central modulators of NK cells function in the tumor microenvironment.

Furthermore, the expression of NK activating genes was positive and significantly correlate with the expression of molecules involved in Th1 signaling, which we already reported to be associated with patients’ favorable outcome [6]. This suggests that, in the tumor microenvironment, the evaluation of collaboration between the innate and adaptive immune systems may be more relevant than single system/cell analysis.

A paradoxical positive association was found between favorable outcome and expression of FOXP3, previously reported to be associated with worse overall survival of patients with breast cancer [51]. This observation is, however, in line with other studies on patients with colon cancer where an association between the infiltration of FOXP3+ T cells and favorable prognosis was evident [52-54]. In addition, emerging evidence suggest that FOXP3 is variably expressed in T cells during activation [55], as well as on tumor cells [56,57]. Finally, activated NK cells may dominate and overcome tumor-induced immune suppression as previously reported by our group [15].

## Conclusions

Results from the present study, together with our recently published work [6], underline the pivotal role played by the innate and adaptive immune systems in the tumour milieu and their coordinated action against cancer progression. Thus, the evaluation of collaboration between the innate and adaptive immune systems may be more relevant than single system/cell analysis. In order to evaluate whether the described results are specific for breast cancer or can be more broadly associated with different cancer types, similar analysis are now ongoing on specimens derived from patients with different tumour types.

The identification of a subtle variation between different tumours in the regulation of molecules mostly associated to NK cells may lead to a better understanding of the intimate relationship between different types of cancer and the immune system.

## Competing interests

None of the authors has a conflict of interest regarding this study.

## Authors’ contributions

All authors read and approved the final manuscript.

## Supplementary Material

Additional file 1: Table S1 Clinical information of the patients and tumour specimens used in each assay. Relapse free outcome was assayed considering the patients’ status at the time of the last follow up (May 2012). Patient VBR7, previously reported as relapse, is confirmed as relapse free. Despite this patient’s outcome variation, the conclusion previously reported [6] sustained based on retrospective repeated analysis. Click here for file

Additional file 2: Figure S1 Canonical pathway based on NK cell–DCs crosstalk at the significance level of 0.001 in the unpaired Student’s t test. Genes highlighted in red or green are upregulated or downregulated, respectively, in relapse-free group of patients. Click here for file

Additional file 3: Figure S2 Canonical pathway based on NK signalling at the significance level of 0.001 in the unpaired Student’s t test. Genes highlighted in red or green are up regulated or down regulated, respectively, in relapse-free group of patients. Click here for file

Additional file 4: Figure S3 A) Expression of FOXP3 in relapse-free and progressing patients; B) Expression of KIR2DL3, KIR3DL3 and KIR2DL2 in relapse-free and progressing patients; C) IHC sections of formalin-fixed paraffin-embedded tumour tissues of breast cancer patients; D) Survival risk prediction analysis based on Age and ER, PR and HER2 status of breast cancer patients. Click here for file

Additional file 5: Table S2 Survival Analysis. Type of univariate test used: Cox proportional hazards model, Wald Statistic. Permutation p-values for significant genes were computed based on 10000 random permutations. Hazard ratio is the ratio of hazards for a two-fold change in the gene expression level. It is equal to exp (b) where b is the Cox regression coefficient. Click here for file
