# Supplementary material for: Molecular signatures mostly associated with NK cells are predictive of relapse free survival in breast cancer patients
Source: J Transl Med. 2013 Jun 12;11:145. doi: 10.1186/1479-5876-11-145 (PMC3694475; doi:10.1186/1479-5876-11-145)
Supplement: Additional file 1: Table S1 — Clinical information of the patients and tumour specimens used in each assay. Relapse free outcome was assayed considering the patients’ status at the time of the last follow up (May 2012). Patient VBR7, previously reported as relapse, is confirmed as relapse free. Despite this patient’s outcome variation, the conclusion previously reported [6] sustained based on retrospective repeated analysis. [file 1479-5876-11-145-S1.doc]

| **Patients** | **Relapse**  **(months)** | **#Relapse-free (months)** | **ER** | **PR** | **HER2** | **Age** | **Stage** | **Gene array** | **qRT-PCR** | **IHC** |
| --- | --- | --- | --- | --- | --- | --- | --- | --- | --- | --- |
| VBR8 | 53 |  | Pos | Neg | Pos | 46 | II | Yes | Yes |  |
| VBR28 | 45 |  | Neg | Neg | Neg | 57 | I | Yes |  | Yes |
| VBR31 | 42 |  | Neg | Neg | Neg | 57 | III | Yes |  |  |
| VBR76 | 9 |  | Neg | Neg | Neg | 29 | I | Yes | Yes |  |
| VBR77 | 32 |  | Neg | Neg | Neg | 41 | II | Yes | Yes | Yes |
| VBR78 | 76 |  | Neg | Neg | Neg | 49 | I | Yes | Yes |  |
| VBR79 | 21 |  | Neg | Neg | Neg | 57 | II |  | Yes | Yes |
| VBR80 | 11 |  | Neg | Neg | Neg | 75 | II | Yes | Yes | Yes |
| VBR04b |  | 70 | Neg | Neg | Neg | 52 | III | Yes | Yes |  |
| VBR10 |  | 62 | Neg | Neg | Neg | 40 | II | Yes | Yes |  |
| VBR12 |  | 62 | Pos | Pos | Pos | 44 | I | Yes | Yes | Yes |
| VBR13 |  | 110 | Neg | Neg | Pos | 39 | I | Yes | Yes | Yes |
| VBR23 |  | 76 | Pos | Pos | Neg | 77 | II | Yes | Yes |  |
| VBR70 |  | 60 | Neg | Neg | Pos | 58 | II | Yes | Yes |  |
| VBR75 |  | 65 | Pos | Neg | Pos | 63 | II |  | Yes | Yes |
| VBR61 |  | 58 | Pos | Pos | Neg | 77 | III |  | Yes | Yes |
| VBR71 |  | 59 | Pos | Pos | Neg | 45 | II |  | Yes | Yes |
| VBR74 |  | 63 | Pos | Pos | Neg | 49 | III |  | Yes |  |
| VBR7* |  | 116 | Pos | Pos | Neg | 64 | II | Yes |  |  |
|  |  |  |  |  |  |  |  |  |  |  |

**Supplemental Table 1. Tumor specimens used in different assays.** #Relapse free outcome was assayed considering the patients’ status at the time of the last follow up (May 2012). Patient *VBR7, previously reported as relapse, is confirmed as relapse free. Despite this patient’s outcome variation, the conclusion previously reported sustained based on retrospective repeated analysis.
