# Supplementary material for: Molecular signatures mostly associated with NK cells are predictive of relapse free survival in breast cancer patients
Source: J Transl Med. 2013 Jun 12;11:145. doi: 10.1186/1479-5876-11-145 (PMC3694475; doi:10.1186/1479-5876-11-145)
Supplement: Additional file 3: Figure S2 — Canonical pathway based on NK signalling at the significance level of 0.001 in the unpaired Student’s t test. Genes highlighted in red or green are up regulated or down regulated, respectively, in relapse-free group of patients. [file 1479-5876-11-145-S3.ppt]

## Slide 1
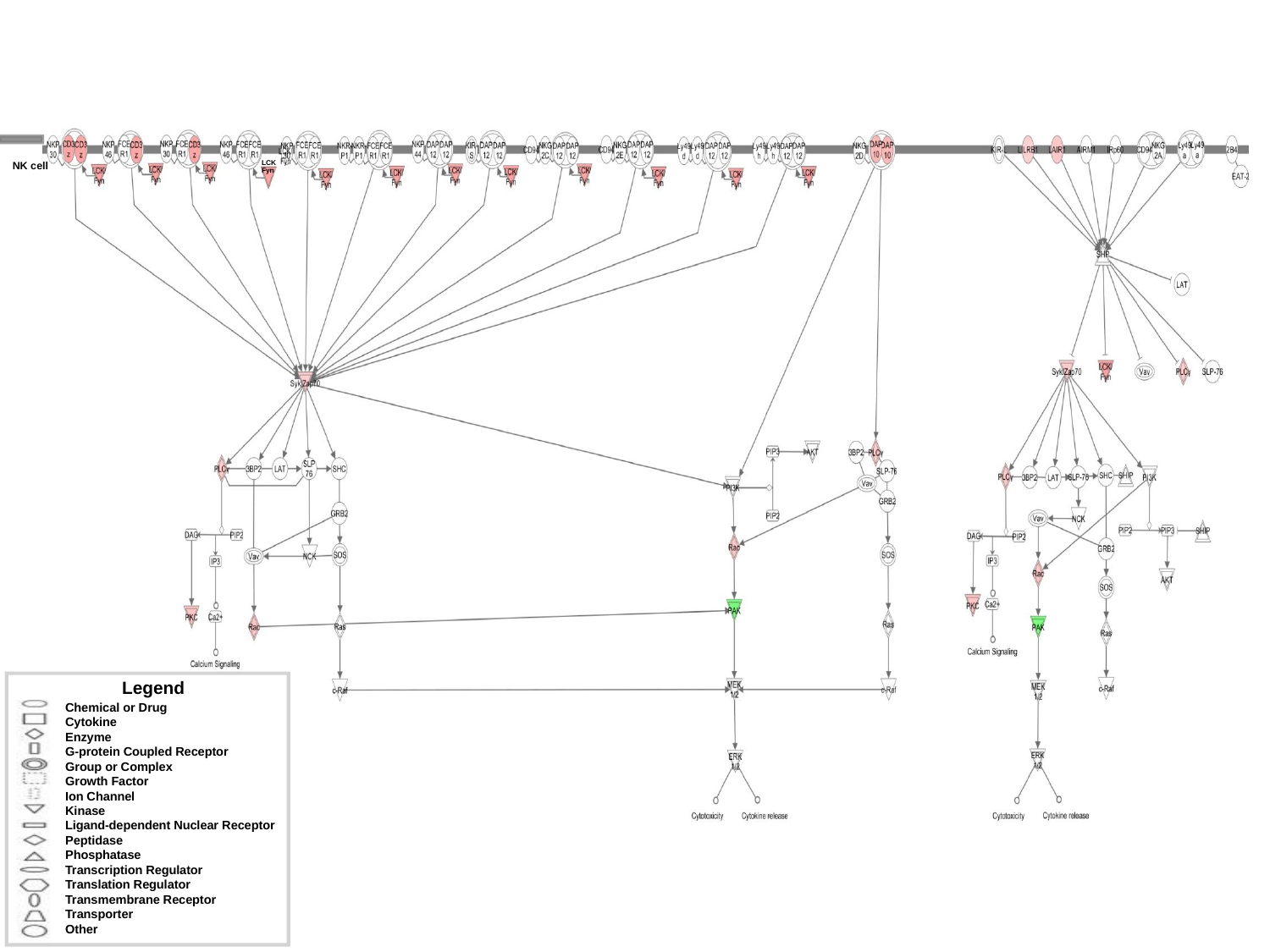

LCK
Fyn
NK cell
Legend
Chemical or Drug
Cytokine
Enzyme
G-protein Coupled Receptor
Group or Complex
Growth Factor
Ion Channel
Kinase
Ligand-dependent Nuclear Receptor
Peptidase
Phosphatase
Transcription Regulator
Translation Regulator
Transmembrane Receptor
Transporter
Other
