# Supplementary material for: Molecular signatures mostly associated with NK cells are predictive of relapse free survival in breast cancer patients
Source: J Transl Med. 2013 Jun 12;11:145. doi: 10.1186/1479-5876-11-145 (PMC3694475; doi:10.1186/1479-5876-11-145)
Supplement: Additional file 4: Figure S3 — A) Expression of FOXP3 in relapse-free and progressing patients; B) Expression of KIR2DL3, KIR3DL3 and KIR2DL2 in relapse-free and progressing patients; C) IHC sections of formalin-fixed paraffin-embedded tumour tissues of breast cancer patients; D) Survival risk prediction analysis based on Age and ER, PR and HER2 status of breast cancer patients. [file 1479-5876-11-145-S4.ppt]

## Slide 1
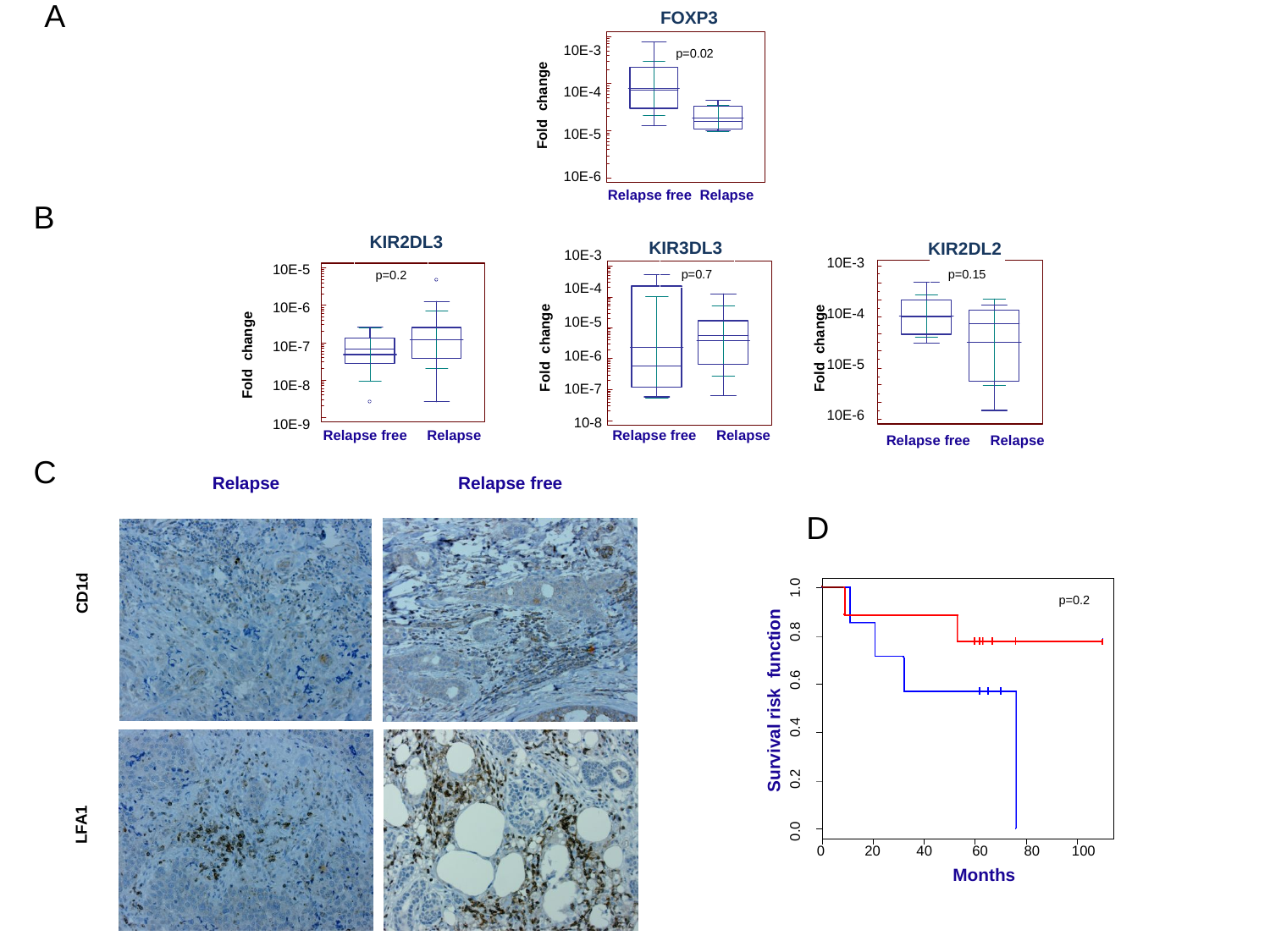

FOXP3
10E-3
10E-4
10E-5
10E-6
A
p=0.02
Fold change
 Relapse free Relapse
10E-3
10E-4
10E-5
10E-6
B
KIR2DL3
KIR3DL3
10E-3
10E-4
10E-5
10E-6
10E-7
10-8
Fold change
KIR2DL2
10E-5
10E-6
10E-7
10E-8
10E-9
KIR2DL3
Fold change
 Relapse free Relapse
p=0.7
p=0.15
p=0.2
Fold change
 Relapse free Relapse
 Relapse free Relapse
C
Relapse
 Relapse free
D
CD1d
p=0.2
Survival risk function
0.0 0.2 0.4 0.6 0.8 1.0
LFA1
0 20 40 60 80 100
Months
