# Supplementary material for: Molecular signatures mostly associated with NK cells are predictive of relapse free survival in breast cancer patients
Source: J Transl Med. 2013 Jun 12;11:145. doi: 10.1186/1479-5876-11-145 (PMC3694475; doi:10.1186/1479-5876-11-145)
Supplement: Additional file 5: Table S2 — Survival Analysis. Type of univariate test used: Cox proportional hazards model, Wald Statistic. Permutation p-values for significant genes were computed based on 10000 random permutations. Hazard ratio is the ratio of hazards for a two-fold change in the gene expression level. It is equal to exp (b) where b is the Cox regression coefficient. [file 1479-5876-11-145-S5.docx]

| **Parametric p-value** | **Permutation p-value** | **Hazard Ratio** | **Gene name** |
| --- | --- | --- | --- |
|  |  |  |  |
| 0.03 | 0.03 | 0.38 | CD 96 |
| 0.05 | 0.04 | 0.40 | NCR3 isoform 1 |
| 0.05 | 0.03 | 0.31 | NCR1 |
| 0.05 | 0.05 | 0.50 | NKG2D |
| 0.14 | 0.14 | 0.60 | CRTAM |
| 0.15 | 0.12 | 0.88 | NCR2 |
| 0.20 | 0.20 | 0.68 | DNAM1 |
| 0.35 | 0.39 | 0.65 | NCR2 |

**Supplemental Table 2. Survival Analysis.** Type of univariate test used: Cox proportional hazards model, Wald Statistic. Permutation p-values for significant genes were computed based on 10000 random permutations. Hazard ratio is the ratio of hazards for a two-fold change in the gene expression level. It is equal to exp(b) where b is the Cox regression coefficient.
